# Surface Modification of a Polyester-Augmented Cellulose Filter for Dehydration of Low-Sulfur Diesel

**DOI:** 10.1021/acsomega.1c01871

**Published:** 2021-07-12

**Authors:** Andrzej Krasiński, Patrycja Jachimczyk

**Affiliations:** Faculty of Chemical and Process Engineering, Warsaw University of Technology, Waryńskiego 1, 00-645 Warsaw, Poland

## Abstract

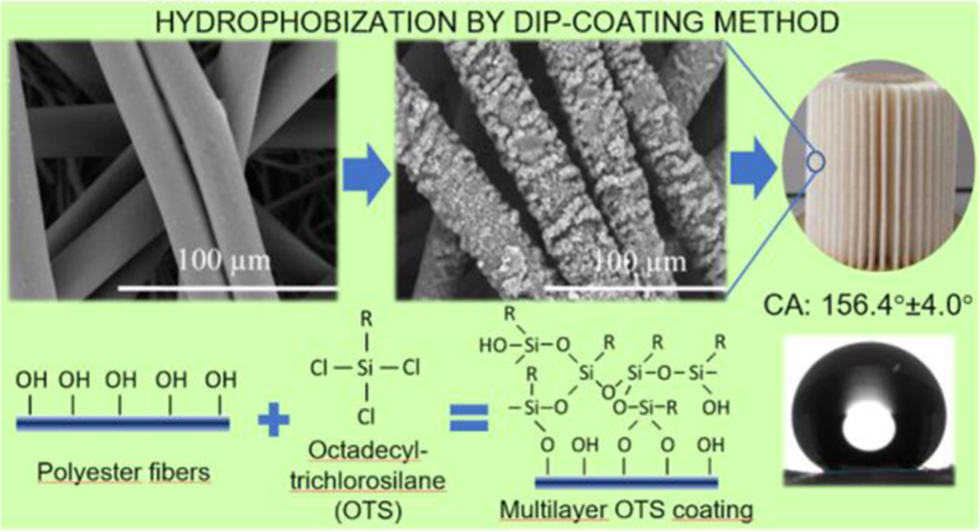

Fuel filters play an essential role in protecting the injection equipment and fuel tanks against corrosion and damage of diesel engines. Diesel fuel separators are typically made of treated cellulose, fiberglass, or mixed fibrous media (composites), modified with various chemicals to obtain a hydrophobic surface. In the presented research, a modification procedure of polyester fibers supported on cellulose using octadecyltrichlorosilane (OTS) has been developed. The OTS coating renders the filter media superhydrophobic with water contact angle and roll-off angle of 156.4 and 4.9°, respectively. The performance of the modified material was verified in dispersed water removal from low-sulfur diesel fuel. High efficiency and a reliable operation in test conditions were confirmed.

## Introduction

According to the statistics of fuel consumption in the transport sector from 1990 to 2018, obtained from 27 countries of the European Union (EU), diesel-powered vehicles were not replaced by gasoline-powered, hybrid, or electric vehicles despite the rapidly developing popularity and technology of the latter.^1^

Diesel fuel consumption in transport overtook motor gasoline consumption in 1998 and was almost 3 times higher in 2018 than gasoline. Over the past 20 years, the demand shift has been dictated mainly by the favorable excise taxes on diesel fuel and the growing medium and heavy-duty transport in the EU.^2,3^ Hence, diesel fuel-driven vehicles still play an important role in this market sector. Nevertheless, improvements are still being introduced, which guarantee the increase of efficiency and reduction of pollutants in the exhaust gases. However, on the other hand, the sophisticated common rail (CR) injection system requires high-purity fuel for reliable operation because they are vulnerable to both solids and free water.

The market demand for high-quality diesel fuel has led to the continuous development of new filtration techniques and separation materials.^4,5^ Diesel filtration problems originate from the introduction of emission limits for pollutants such as carbon monoxide (CO), nitrogen oxides (NOx), particulate matters (soot and ash), and unburned hydrocarbons (HC) for diesel engines.^6,7^ The regulations introduced due to environmental concerns and human health safety lead to the development of diesel engine technology, such as high-pressure CR fuel injection systems.^8,9^ Additionally, due to the proven detrimental effect of high sulfur level in diesel fuels on engine parts and the emission, a progressive decrease of sulfur content in diesel fuels was imposed by regulatory agencies. Consequently, ultra-low-sulfur diesel (ULSD) with a maximum of 10 parts per million sulfur (ppm), regulated in the EN 590:2009 standard, was introduced gradually on the European market. A similar tendency was observed in North America due to the diesel fuel standard (ASTM D975-20c) introduced by the United States Environmental Protection Agency (U.S. EPA). The substantial reduction of sulfur is typically obtained in the deep desulfurization process by hydrogenation, but it leads to the loss of fuel lubricity.^10,11^ To maintain the lifetime of diesel engine parts, diesel fuel is doped with a composition of additives, such as, lubricity enhancers, cetane number improvers, antistatic additives, antioxidants, and corrosion inhibitors, among others.^12^ Some of these additives tend to adsorb at the interphase and become efficient stabilizers for water droplets in diesel fuel. This change of fuel chemistry contributed to the failures of separation and coalescence materials used for diesel filtration, including water removal.^13^

The vehicles powered by diesel fuel can be equipped with a single primary filter or a system of primary and secondary filters. The primary filters are widely used in the fuel supplying system to remove most solids (typically larger than 10 μm), waxes, and water. They are placed on the suction side of the high-pressure fuel pump. However, the secondary fuel filters are installed to block the fine particles passing the primary filter, and they are mounted downstream of the fuel pump. Due to different applications in the fuel supplying system, the filtration media used in both types of filters should have carefully designed surface and structural properties.

The subject of this research is the development and experimental verification of a new filtration material with dedicated use in primary filters. The effective performance of this separation medium relies on stopping the water on the upstream side of filter media due to capillary forces, efficient detachment of the collected water droplets from the superhydrophobic surface, and the low pressure loss for the flow of diesel fuel through the relatively small pore size of the material.^14^

Generally, the strategies used to attain hydrophobicity rely on decreasing the surface energy, creating surface roughness, or both of them, depending on the applied deposition method. Surface hydrophobization techniques comprise such processes as chemical vapor deposition,^15^ electro-spraying,^16^ nanoparticle deposition,^17^ dip-coating,^18^ sol–gel processing,^19,20^ layer-by-layer (LbL) deposition,^21^ plasma treatment,^22,23^ spraying,^24^ etching,^25^ and others.^26,27^ Next to the deposition methods, the chemical compounds used during the material functionalization play an important role. The most widely used modifiers for hydroxylated materials such as silicon oxide,^28,29^ aluminum oxide,^30^ glass,^31^ wood,^32,33^ and cellulose-based papers^18,34^ seem to be silanes. Despite the popularity of silane, octadecyltrichlorosilane (OTS) usage for deposition on polymeric nonwoven materials is not well studied or described in the literature.^35^

In the presented work, a deposition method of octadecyltrichlorosilane (OTS) on the outer layer of filtration media, consisting of polyester fibers, was developed. Therefore, the polyester fibers, which are included to increase the dust-holding capacity of the media after surface modification, gain a highly hydrophobic surface, which repels water droplets and enables collecting them on the upstream side of the filtration material. In the final part, the modification procedure was scaled up, and the separator’s performance in the experiment of dispersed water removal from ultra-low-sulfur diesel fuel was verified. The influence of operating conditions such as face velocity and inlet water concentration on separator performance was investigated and discussed.

## Materials and Methods

In this work, the commercial grade of filter media 350 H/SM50 from Ahlstrom was used. The essential filtration layer consists of cellulose fibers augmented with two polyester layers: a spun-bond polyethylene terephthalate layer and a melt-blown polybutylene terephthalate layer. The polyester fibers constitute the additional prefiltration layers laid on the cellulose support. The properties of the reference separation material are given in Table 1.

**Table 1:** Specification of the Reference Separation Media Used in Experiments

material property	value
thickness	750 μm
grammage	280 g/m^2^
resin content	15%
average diameter of polyester fibers	4.9 μm
bubble test—1BP	500 mmWC
maximum pore size^a^	19 μm

a Value obtained using mathematical correlation.

### Surface Modification Procedure—Coating with Octadecyltrichlorosilane (OTS)

The modification of the reference filtration material was performed using octadecyltrichlorosilane (OTS). The silane molecules form thick layers attached to the surface in an ordered manner (self-assembled monolayers). The main reactions considered during the process of surface modification are hydrolysis and condensation. Condensation can occur between the organosilicon coupling molecule and the −OH group exposed on the polyester or cellulose surface (heterocondensation) or between coupling molecules (homocondensation). The latter one leads to the formation of Si–O–Si bonds.^36^ Therefore, the process is governed by the balance between hetero- and homocondensation, which depends on modified material properties and the water concentration.^37^ However, at a high water concentration, homocondensation can lead to the polymerization of the organosilicon molecules. Thus, the presence of water vapor in the ambience or adsorbed on the surface of oxide can significantly affect the morphology of the coating layer.^38^

Due to the moisture sensitivity of OTS, the modifications were carried out in a closed container. As the OTS solvent, *n*-hexane (analytical grade) was used. The water concentration in *n*-hexane, measured by the Karl Fischer coulometric method, was equal to 34.8 ± 5.8 mg/L. Before processing, all samples were dipped in *n*-hexane for 1 h and dried in the convective dryer at 50 °C for 1 h to remove the remaining moisture from the material. The modification process was divided into two parts: (I) deposition of OTS on the reference material by dip-coating (1% v/v OTS in *n*-hexane for 1, 2, or 4 h in ambient temperature) and (II) drying in the convective dryer at 50 °C for 1 h or even up to 8 h in the case of pleated filters (full-scale elements for testing).

The abbreviations and a brief description of the materials are provided in Table 2.

**Table 2:** Details of the Materials Obtained by the Dip-Coating Method

material symbol	OTS solution concentration, % v/v	dipping time, hours
C	reference sample (untreated)	
C-OTS-1	1	1
C-OTS-2	1	2
C-OTS-4	1	4

### Characterization of the Treatment Effect on Properties of the Separation Material

#### Physicochemical Properties

The presence of OTS deposits on the substrate was confirmed by Fourier transform infrared spectroscopy (FTIR) analysis using a Nicolet iS10 spectrometer from Thermo Scientific in attenuated total reflection (ATR) mode. This technique enables monitoring the C–H stretching vibration at wavenumbers around 2850 and 2920 cm^–1^, assigned to methylene groups present in the long hydrocarbon chain of OTS (−C_18_H_37_).

#### Wettability of the Filtration Material

In this work, static and dynamic contact angle measurements are used to characterize the surface properties of separation materials. The mentioned methods are dedicated to flat, smooth, and nonporous surfaces. Despite criticism, the method is also commonly used in research of fibrous materials, such as filtration media. The net values of contact angle are questionable, but they can be used for comparison between materials of the same or similar structure and can provide information about the surface properties.

In this research, measurements of water contact angles in air were carried out. The dynamic advancing and receding contact angle (ARCA) method was used to determine the hysteresis of contact angles. The ARCA method relies on shape analysis of droplets during dosing and withdrawing of the test liquid on a flat horizontal sample. The difference between advancing (the highest value) and receding (the lowest value) contact angles is the hysteresis of contact angles. The same dynamic contact angles can be observed when the droplet slides or rolls off along the sloped surface. The minimum inclination angle for the droplet movement provides information on the self-cleaning properties of the surface from the collected water droplets. In this work, the static method was involved, i.e., the droplet was placed on the surface, and the goniometer base was tilted. The angle at which the droplet starts moving accounted for the sliding angle.

Measurements of static contact angle, sliding angle, and dynamic contact angle hysteresis for water droplets were carried out using a Dataphysics OCA 25 goniometer equipped with a tilting base. The sliding angle was determined for 50 μL water droplet volume.

#### Surface Morphology of Separation Media

The structure of deposits formed on the fibers of the filtration material was investigated using scanning electron microscopy (SEM) and atomic force microscopy (AFM). These techniques provide information about the character of coating (homogeneous, continuous, rough, or smooth) at the microscopic level and also help to explain the observed results of the materials’ wettability measured in the macroscale. The scanning electron microscopy images were obtained using a Hitachi TM-1000 scanning electron microscope. The analysis of surface topography was carried out using a MultiMode Nanoscope 8 (Bruker) atomic force microscope. In all experiments, ACSTA probes (AppNano) were applied (nominal spring constant 7.8 N/m, nominal frequency 150 kHz). All measurements were performed in ambient conditions on dry samples. The analysis of sample surface scans with the AFM enabled determining the values of three parameters: average roughness (*R*_a_), root-mean-square roughness (*R*_q_), and maximum profile height (*R*_t_), which characterize the geometry of the surface structure.

#### Verification Method of the Performance of the Modified Separation Material

**Figure 1:**
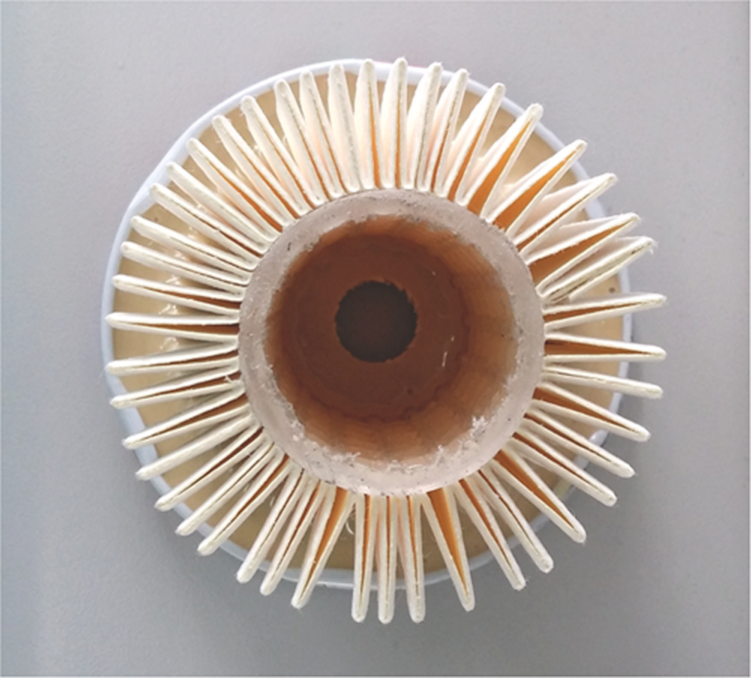
Top view of the pleated separator (without the end cap).

The key point of this research was the verification of the material’s separation performance in the experiment of water removal from commercial grade diesel fuel from PKN Orlen. For this purpose, the full-scale pleated separators with an out-to-in flow direction were constructed (Figure 1). The separators had a surface area of 0.086 m^2^ and detailed dimensions were as follows: the total element height 65 mm, the height of pleats 15 mm, and the number of pleats 44.

**Figure 2:**
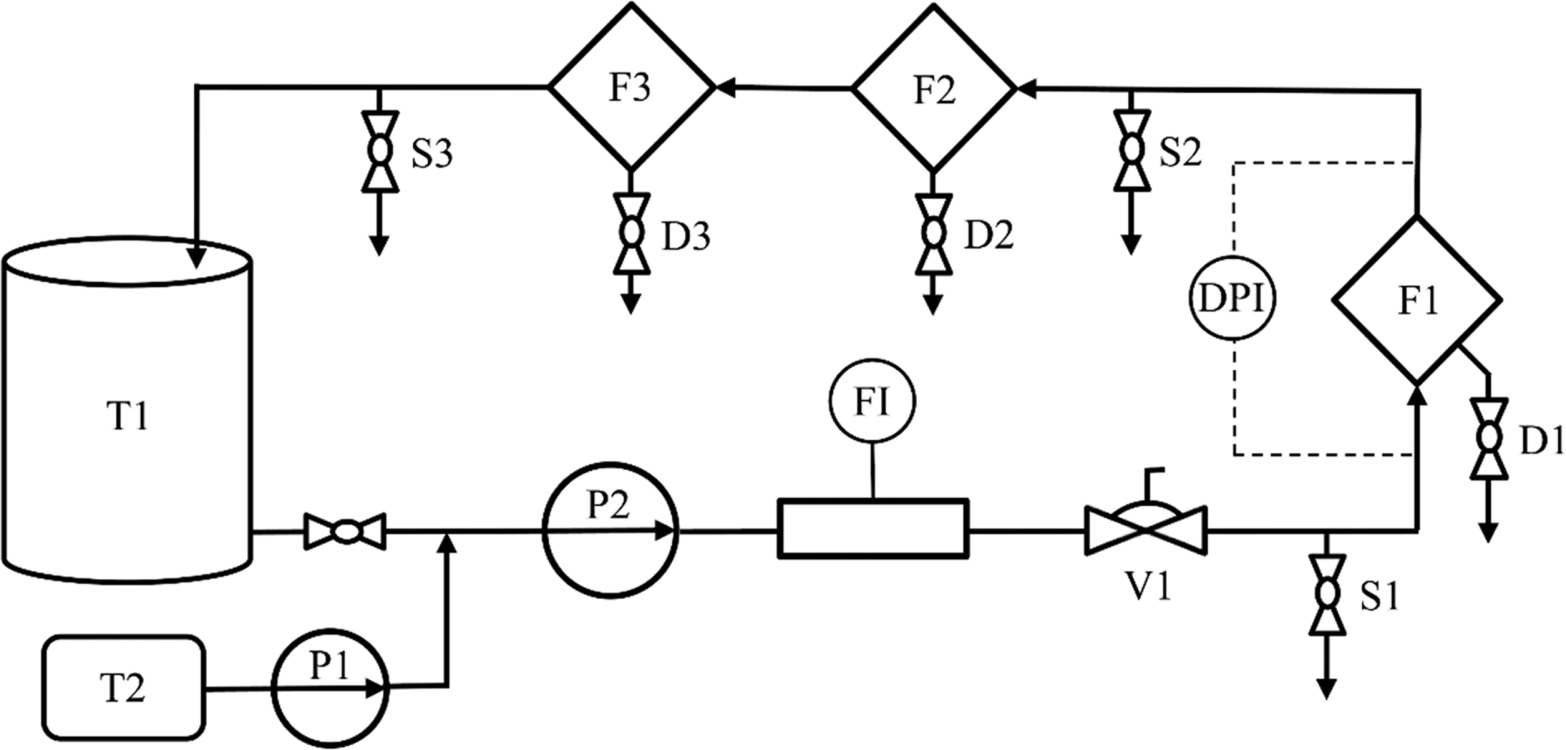
Scheme of the experimental setup: T1—diesel fuel tank, T2—water tank, F1—test filter, F2 and F3—purifying filters, P1—membrane dosing pump, P2—centrifugal pump, FI—mass flowmeter, V1—membrane valve for flow control, DPI—differential pressure sensor, S1, S2, and S3—sample points, D1, D2, and D3—water drains.

The experiments of water separation were carried out in the test rig, schematically presented in Figure 2. The system is equipped with a centrifugal pump P2, which circulates the fuel in the system. Additionally, pump P2 generates the emulsion in a single pass of water through its impellers. The pump speed determines the water droplet size distribution. In all experiments, 20% of maximum pump speed (max. 2835 rpm) was set up. Water is dosed to the system upstream of pump P2 by the membrane dosing pump P1. The liquid circulates from tank T1 through a glass filter housing F1, where the test filter is mounted. The commercial filters F2 and F3 purify the diesel fuel returning to the tank from any remaining water. The membrane valve V1 is used for flow control. Sample points S1 and S2 are used to monitor the total water concentration in the inlet and outlet streams, respectively. Thus, the sample valve S3 enables control of the content of dissolved water in diesel fuel (assuming that no free water goes through the additional filters F2 and F3). During the 150 min of test time, the pressure drop and the water concentration using the Karl Fischer coulometric method (Titrando 851 from Metrohm) were determined every 30 min. All parameters that characterize the dispersion and test conditions are listed in Table 3.

**Table 3:** Specification of Experimental Conditions

parameter	value
flow rate	46–92 l/h
face velocity	0.15–0.30 mm/s
inlet water concentrations	1000, 2500 or 5000 mg/L
water droplet size	30.8 ± 23.5 μm (avg. ± std. dev.) 3.9–131.6 μm (min.–max.)
interfacial tension (pendant drop method)	15.8 ± 0.2 mN/m (avg. ± std. dev.)

## Results and Discussion

### FTIR Spectroscopy

**Figure 3:**
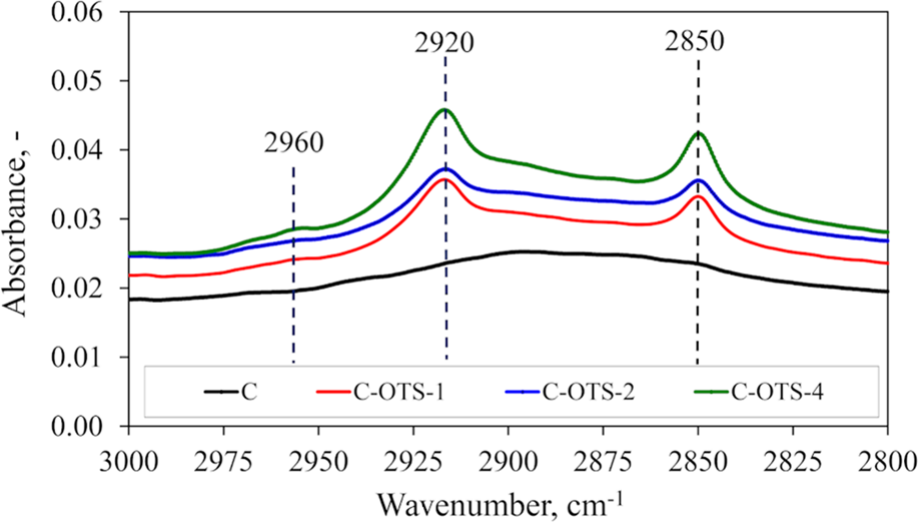
FTIR spectra showing the CH*_x_* region (range 3000–2800 cm^–1^) of the separation material covered by OTS for various dipping times: 1 h (C-OTS-1), 2 h (C-OTS-2), and 4 h (C-OTS-4) in comparison to the reference material (denoted as C).

The spectra of absorption peaks at wavenumbers ranging from 3000 to 2800 cm^–1^ of the separation material covered by OTS for various dipping times are shown in Figure 3. The visible asymmetric and symmetric stretching peaks of CH_2_ at around 2920 and 2850 cm^–1^, respectively, confirm the adsorption of alkyl groups from OTS onto the material. Moreover, the presence of the asymmetric stretching peak of CH_3_ at about 2960 cm^–1^ is visible, especially for the material treated for 4 h. The increasing intensity of peaks with the dipping time of the material in OTS solution is also observed. It can indicate a more dense covering on the material or the formation of more OTS cross-linked coating layers due to extended silanization.

### Static and Dynamic Contact Angles and Roll-Off Angle with Water

**Figure 4:**
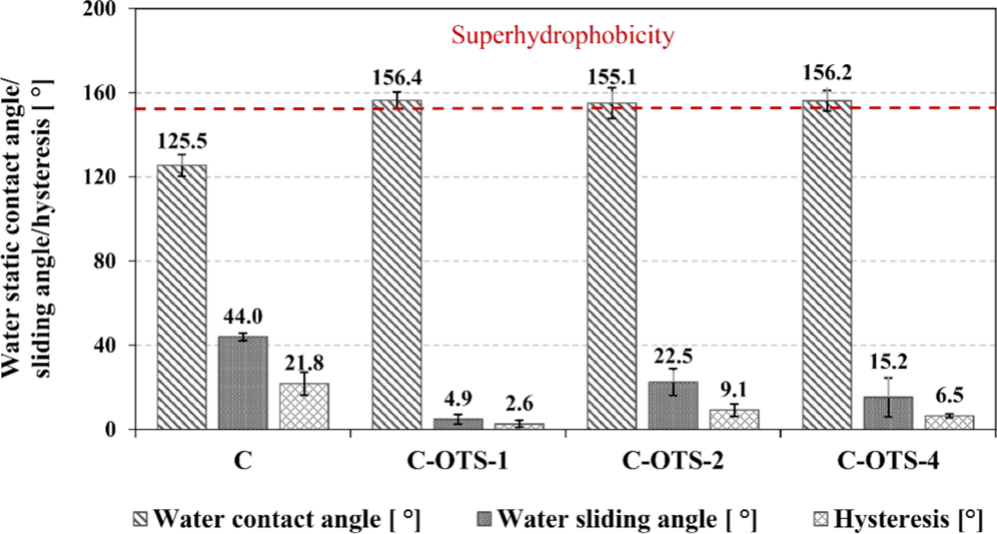
Water static contact angle, sliding angle, and hysteresis obtained for OTS-modified materials depending on the treatment time: 1 h (C-OTS-1), 2 h (C-OTS-2), and 4 h (C-OTS-4) in comparison to the reference material (denoted as C).

Due to the self-assembling ability and low critical surface tension of OTS (18.2 mN/m),^31^ it was feasible to obtain fiber coatings characterized by superhydrophobic properties. The obtained results of wettability for the modified samples are shown in Figure 4. Water static contact angles higher than 155° for all C-OTS samples and enhanced roll-off properties were obtained for the treated media compared to the reference material (C). The higher the water contact angle value, the stronger the component of capillary force preventing the water from entering the hydrophobic structure and passing through the filtration barrier (for the same pore size). This guarantees the reliability of operation and means that a higher face velocity can be used for the same structure of the filtration material, which directly increases the throughput (or size) of the filter. The low value of the water sliding angle (4.9° for C-OTS-1) or a small hysteresis of the dynamic water contact angles (2.6° for C-OTS-1) reflects the better self-cleaning ability of the surface to repel water during the process. This parameter has a direct effect on the extension of filter operation time.

Additionally, the obtained results show the lack of significant influence of the treatment time on the wettability of the material in the analyzed range. The adsorption kinetics of OTS molecules is determined by the availability of −OH groups on the surface. The density of −OH groups decreases with the increase of dipping time. Therefore, the adsorption kinetics of OTS molecules slows down.

### Morphology of OTS-Coated Separation Media

**Figure 5:**
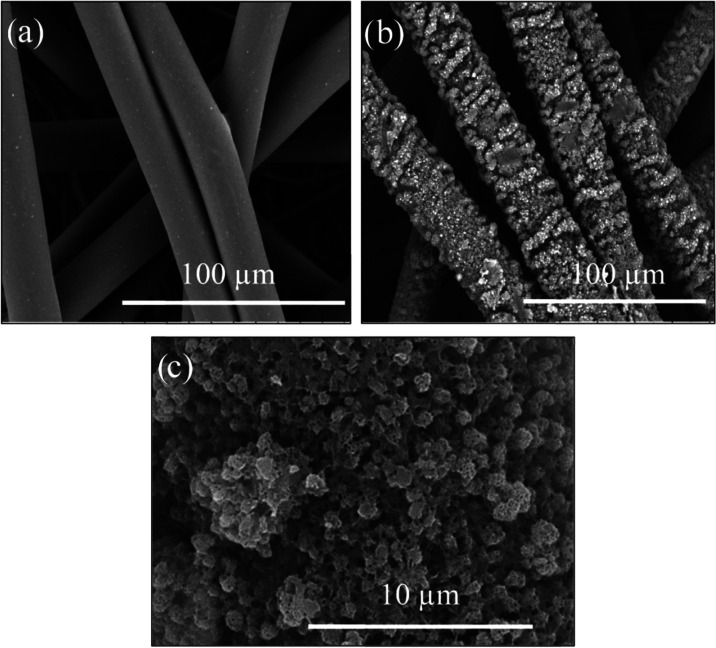
Sample SEM images showing the morphology of the fiber surface of (a) untreated material and (b, c) material with OTS coating for the treatment time of 1 h.

Next to the properties that define the filtration materials’ wettability, the surface morphology helps to verify the uniformity and the detailed structure of prepared coatings. In Figure 5, the SEM images of C-OTS sample for 1 h of treatment time are presented. Rough surfaces of the fibers are visible in the SEM image. The OTS aggregates exhibit considerable self-organization, and they appear on the surface as “islands” with a highly porous structure. The observed particulate nanostructure of deposits arises from a relatively high concentration of OTS solution, which enables the formation of the cross-linked multilayers due to hydrolysis and condensation of silane.^31,34^

**Figure 6:**
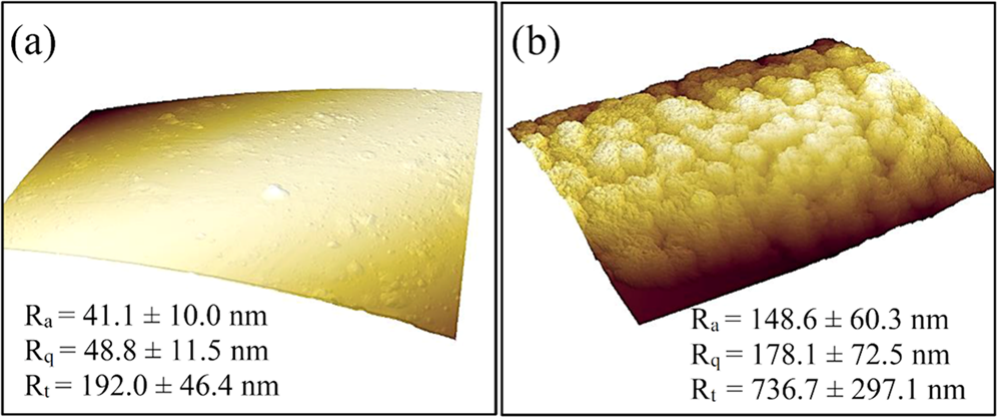
AFM 3D fiber surface images of (a) the reference material (C) and (b) the material dipped in OTS solution for 1 h (C-OTS-1).

Additionally, the C-OTS-1 and reference C samples were analyzed by atomic force microscopy. The surface morphology obtained from AFM is shown in Figure 6, where a very smooth reference fiber is compared to the rough multilayer structure obtained after 1 h of the wet growth conditions of OTS. The complete surface coverage of fibers by a uniform layer has been obtained. The value of the parameters determined based on the AFM analysis, roughness average (*R*_a_), root-mean square roughness (*R*_q_), and maximum profile height (*R*_t_), are complementary for the SEM images. The *R*_a_ value for the C-OTS sample is 148.6 nm, about 4 times higher than the *R*_a_ of the reference C sample (about 41 nm). The deviation around the average value is also higher. A similar tendency was observed for *R*_q_ and *R*_t_ parameters for both samples.

### Experimental Results of Water/Diesel Fuel Separation

**Figure 7:**
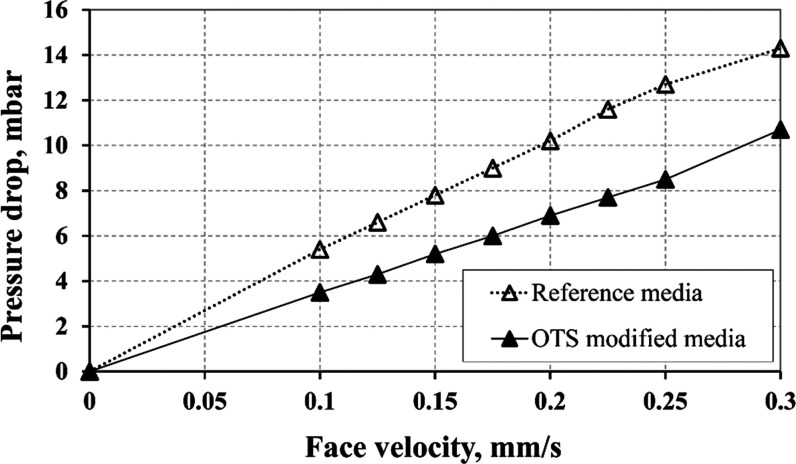
Flow characteristics of the tested separation media for one-phase flow (i.e., for pure diesel fuel).

Based on the wettability and surface morphology results, the material C-OTS-1 was qualified for experiments of diesel fuel dewatering. The test results of separation for the modified filter media were compared with those of the reference (unmodified) structure. In Figure 7, the flow characteristics of the examined filters for clean diesel fuel are presented. It appeared that the application of surface modification reduced the pressure drop, although the growth of some solid forms on the fibers was observed. This phenomenon is counterintuitive but can be explained by the alleged increase in the oleophilicity of the surface. In such a case, stronger surface interactions enhance the capillary transport of diesel throughout tight passages (pores), thus reducing the overall drag force for the flow.

Pleated separators were used in the experiments to increase the filtration area and to keep the size of the filter very compact (suitable for the housing size and the flow rates). The filtration area was kept constant in all experiments, while the face velocity on the inlet ranged from 0.15 to 0.3 mm/s. The lower value of linear velocity corresponds to values advised in the specification of commercial separators for the nominal flow rate. In this area of filtration technology, effort is being made to preserve the high separation efficiency at a high velocity. This enables the reduction of the separator size (or increases the flow rate for the fixed filtration area).

**Figure 8:**
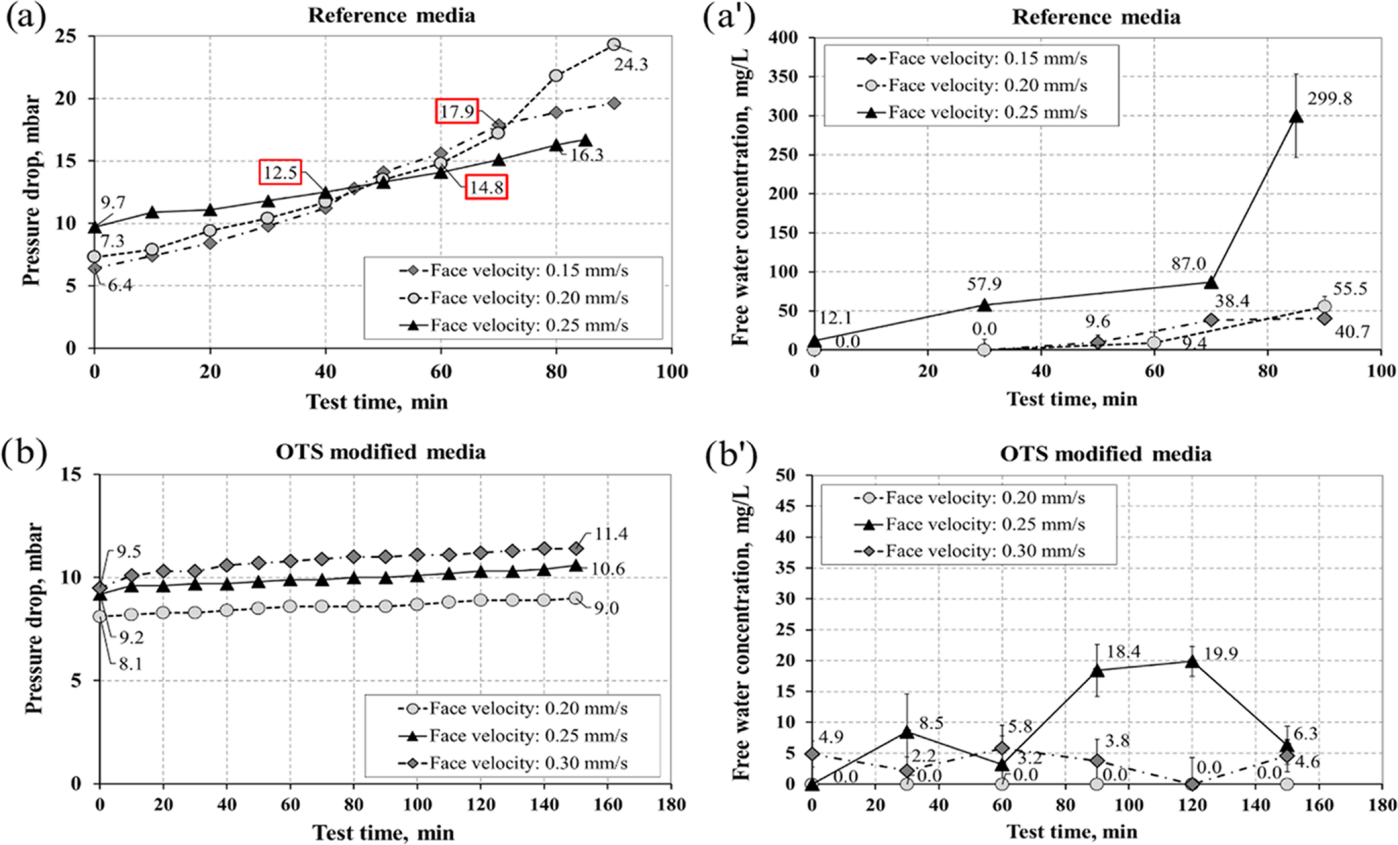
Effect of the face velocity on pressure drop and free water concentration on the outlet obtained for (a, a′) reference media (b, b′) OTS-modified media in experiments of diesel fuel dewatering; inlet water concentration: 2500 mg/L.

**Figure 9:**
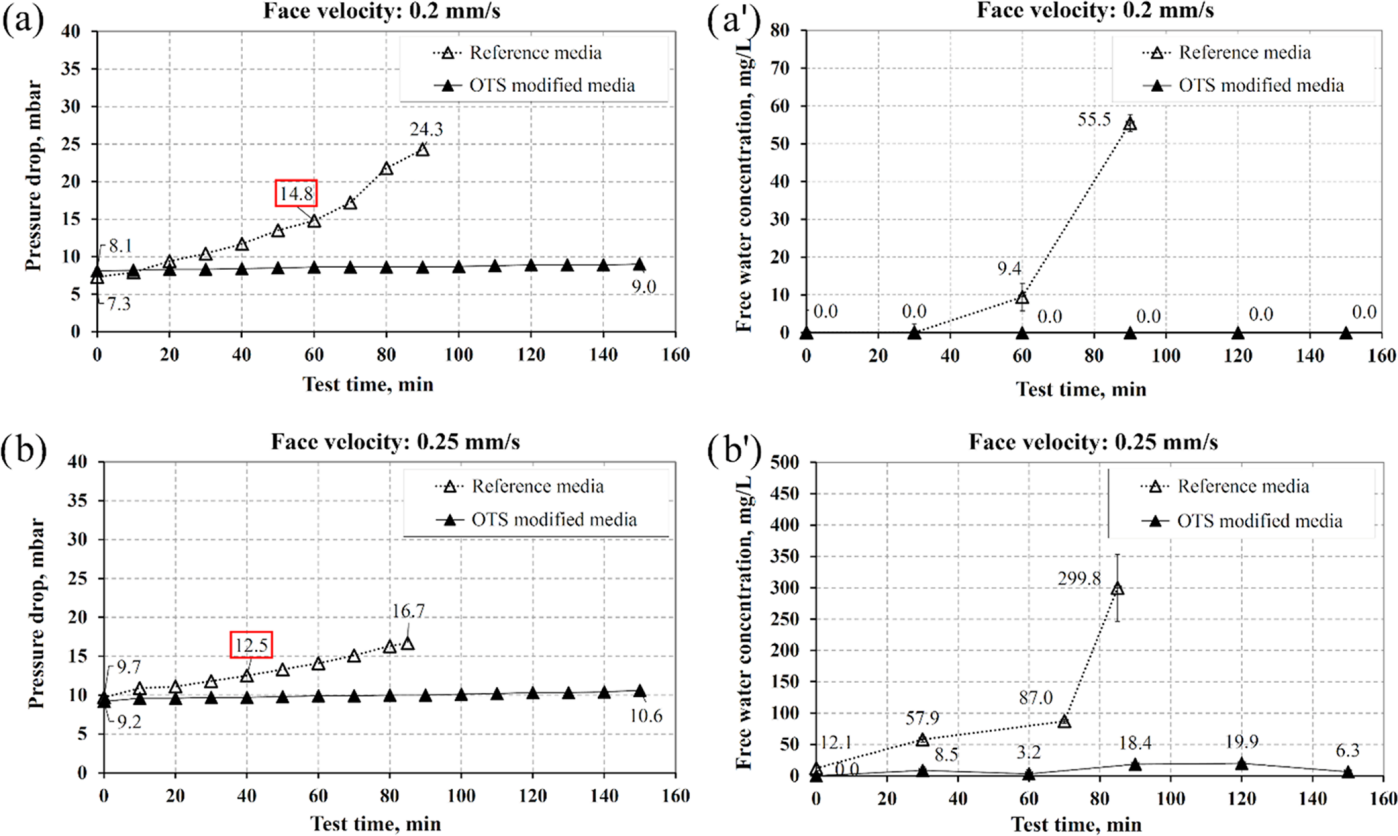
Comparison of pressure drop (a, b) and free water concentration (a′, b′) obtained for the reference and OTS modified filters for the face velocity: 0.2 and 0.25 mm/s; inlet water concentration: 2500 mg/L.

**Figure 10:**
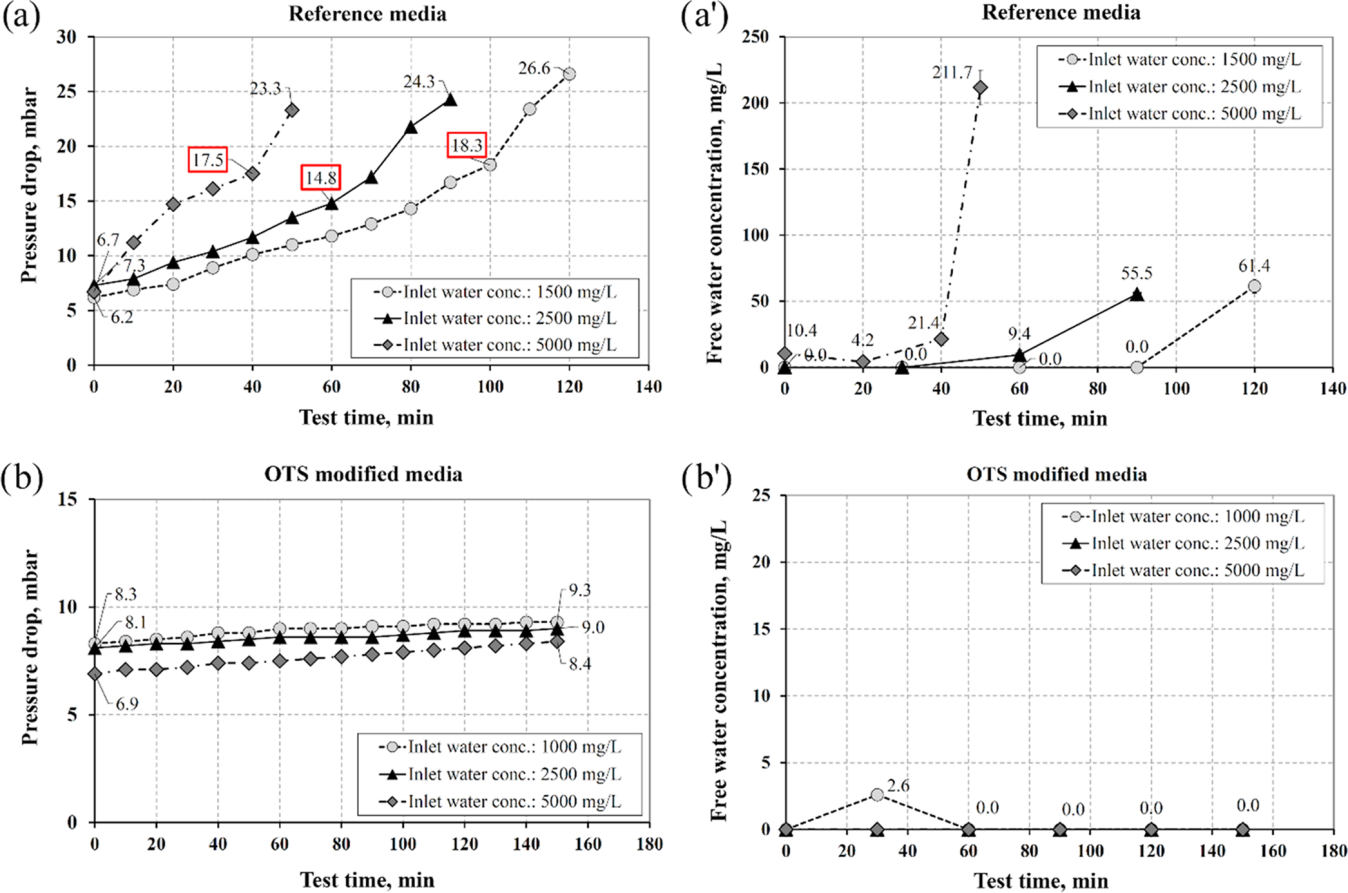
Effect of the inlet water concentration on the pressure drop and the free water concentration on the outlet obtained for (a, a′) reference media and (b, b′) the OTS-modified media; face velocity: 0.2 mm/s.

The results of diesel fuel dewatering for the reference and the OTS modified filters are presented in Figure 8. The pressure drop during the test time was recorded for three linear velocities (Figure 8a,b). The free water concentration was determined by KF titration for downstream of the filter (Figure 8a′,b′). The purified fuel stream was visually inspected during the experiment to capture the moment of water breakthrough. If the water droplets were observed downstream the filter, the fuel sample was taken for the measurement, and the experiment was interrupted. The nature of the process is that the separator completely retains the water upstream of the hydrophobic structure as long as it operates efficiently. However, when the pump impeller creates a dispersion, a broad size distribution of water droplets is obtained, and tiny droplets are also present in the dispersion (contrary to the standard test method ISO 16332). In such a case, the free water concentration on the outlet can be slightly higher than zero and stabilize at this level. However, when the breakthrough occurs, i.e., the water accumulates and starts penetrating through the structure, its concentration in the filtrate rapidly increases. It is not critical to determine the water concentration in the fuel after the breakthrough takes place—there is surely a drastic jump in the water concentration once it happens. It is more important to estimate at what value of the pressure drop it begins, which directly corresponds to the capillary forces. In Figures 8a,b, 9a,b, and 10a,b, the labels on the dP vs time curves denote the initial and final pressure drop values measured during the filtration experiments. The pressure drop values inside a frame correspond to filter breakthrough.

The obtained results confirm an increase of the initial and the final pressure drop due to the increase of linear velocity for both filters (Figure 8). The pressure drop for the unmodified filter increased faster, and depending on the velocity, the water penetration was observed between 12.5 and 17.9 mbar. The efficient operation of the reference filter was interrupted due to filter breakthrough after 40, 60, or 70 min for face velocities 0.25, 0.20, or 0.15 mm/s, respectively. The OTS-coated filter performed with high efficiency for the entire test time (150 min) with only a tiny increase of the pressure drop. It can be summarized that the surface treatment has a significant influence on the steady-state dP value. These horizontal dP vs time profiles at relatively low dP values correspond to the excellent water drainage ability of the separator (no water accumulation) and confirm its good self-cleaning properties.

The above results obtained for the reference and modified filters for face velocities equal to 0.2 and 0.25 mm/s are combined and compared in Figure 9.

Additionally, the influence of the inlet water concentration on the separator operation was investigated. The obtained results clearly show the opposite performance of examined filters when the inlet water concentration increased (Figure 10). In the case of the reference filter, the highest value of inlet water concentration (5000 mg/L) led to the fast filter breakthrough (approx. after 40 min, as shown in Figure 10a), while the performance of the modified filter was better—it operated effectively with a low pressure drop at high inlet water concentration. The observed tendency can be explained by a higher collision rate of inflowing droplets with the deposited ones. The collisions promote the movement of the droplets collected on the surface and their detachment from the low-adhesion surface (Figure 10b).

## Conclusions

In the presented work, a simple and relatively easy method for creating a coating on polyester filter fibers suitable for diesel fuel dewatering has been developed. The obtained surface was characterized by a very high water contact angle, i.e., the surface of treated filters became highly hydrophobic. Moreover, the low sliding angle for water droplets placed on the surface confirmed its low adhesion properties, which in the filtration process can be a measure of its self-cleaning ability—this hypothesis was experimentally confirmed. The OTS-modified filters provided the best results in experiments of water separation from diesel fuel. In such a process, water is stopped on the surface, where it accumulates. However, due to the reduction of water adhesion to the filter, no significant dP increase was observed during the test time. The droplets were easily detached from the hydrophobic surface. In such a case, the dP did not exceed the capillary pressure, and the water did not penetrate through the hydrophobic barrier. The proposed modification can be considered as a promising method for scaled-up applications due to its simplicity. The OTS-based particles are inert and stable in contact with a range of process fluids. No detrimental effect of the coating material has been observed during the research. However, its suitability for fuel processing has to be further investigated.
